# Comparative analysis of the acute response of the trout, *O. mykiss*, head kidney to *in vivo *challenge with virulent and attenuated infectious hematopoietic necrosis virus and LPS-induced inflammation

**DOI:** 10.1186/1471-2164-9-141

**Published:** 2008-03-26

**Authors:** Simon MacKenzie, Joan C Balasch, Beatriz Novoa, Laia Ribas, Nerea Roher, Aleksei Krasnov, Antonio Figueras

**Affiliations:** 1 Unitat de Fisiologia Animal, Departament de Biologia Cellular, de Fisiologia i d'Immunologia, Universitat Autonoma de Barcelona (UAB), Barcelona, Spain; 2 Instituto de Investigaciones Marinas, CSIC, Vigo, Spain; 3 Imperial College Faculty of Medicine, Dpt of Infectious Disease Epidemiology, London, UK; 4 Departament de Fisiologia, Facultat de Biología, Universitat de Barcelona, Barcelona, Spain; 5 Akvaforsk, Institutt for akvakulturforskning, Aas, Norway

## Abstract

**Background:**

The response of the trout, *O. mykiss*, head kidney to bacterial lipopolysaccharide (LPS) or active and attenuated infectious hematopoietic necrosis virus (IHNV and attINHV respectively) intraperitoneal challenge, 24 and 72 hours post-injection, was investigated using a salmonid-specific cDNA microarray.

**Results:**

The head kidney response to i.p. LPS-induced inflammation in the first instance displays an initial stress reaction involving suppression of major cellular processes, including immune function, followed by a proliferative hematopoietic-type/biogenesis response 3 days after administration. The viral response at the early stage of infection highlights a suppression of hematopoietic and protein biosynthetic function and a stimulation of immune response. In fish infected with IHNV a loss of cellular function including signal transduction, cell cycle and transcriptional activity 72 hours after infection reflects the tissue-specific pathology of IHNV infection. attIHNV treatment on the other hand shows a similar pattern to native IHNV infection at 24 hours however at 72 hours a divergence from the viral response is seen and replace with a recovery response more similar to that observed for LPS is observed.

**Conclusion:**

In conclusion we have been able to identify and characterise by transcriptomic analysis two different types of responses to two distinct immune agents, a virus, IHNV and a bacterial cell wall component, LPS and a 'mixed' response to an attenuated IHNV. This type of analysis will lead to a greater understanding of the physiological response and the development of effective immune responses in salmonid fish to different pathogenic and pro-inflammatory agents.

## Background

The orchestration of a successful immune response to infection requires an integrated tissue response coordinated by specific cytokine and chemokine release. Pathogen-specific immune responses are coordinated and dependent upon the activation of specific pathogen recognition receptors (PRRs), molecular moieties present upon sub-sets of leukocytes such as macrophages or dendritic cells. PRRs respond to pathogens or their PAMPs (*P*athogen *A*ssociated *M*olecular *P*atterns) by the initiation of distinct transcriptomic programmes which will dictate the cellular/tissue response [1-3]. In mammals different transcriptional programmes have been identified by microarray analysis for specific PAMPs (viral, bacteria and yeast) by both macrophages and dendritic cells which initiate the immune response by secreting molecules such as pro-inflammatory cytokines [4,5].

The availability of salmonid-specific gene chips [6-8] has provided the means to begin to characterise the salmonid immune response at a global gene level both *in vitro *and *in vivo*. This technology will provide a deeper understanding of overall cellular and tissue processes during immune activation. A number of recent reports concerning PAMP recognition [9], activated macrophage transcriptomics [8] and immunomics [10-16] and genome-wide surveys [17] show that fish and fish macrophages clearly respond differentially to different pathogens. This therefore should lead to different physiological/immunological responses *in vivo *upon which the survival of the organism is based.

The head kidney or anterior kidney located posterior to the cranium, is the central hematopoietic organ in salmonids and other fish species. In addition, it contains adrenalin-producing chromaffin cells and also plays a major endocrine role in secretion of cortisol, the major glucocorticoid and mineralocorticoid in fish [18-21]. The head kidney can thus integrate the neuro-immuno-endocrine milieu in normal and pathological states. However, few global gene regulation studies concerning the molecular regulation of head kidney function during infection or PAMP stimulation in salmonids [15] have been reported although many studies have used this tissue as a primary source of macrophage-like cells for studies on the activation of the immune system [22,23].

The *Novirhabdovirus *infectious hematopoietic necrosis virus (IHNV) is probably one of the most important fish viral pathogens, responsible for great mortalities in farmed salmonids [24,25]. As for all the *Rhabdoviridae*, the genome of IHNV consists of a single-stranded negative-sense RNA which has been entirely sequenced [26,27]. Their genome codes for five structural proteins: a nucleoprotein (N), a polymerase-associated protein (P), a matrix protein (M), an RNA-dependent RNA polymerase (L) and a surface glycoprotein (G) responsible for immunogenicity [28-30]. An additional gene, only present in some fish rhabdoviruses, located between the G and L genes, encodes a non-structural protein NV, whose putative role in virus replication remains to be fully evaluated [31] but appears to be linked to viral growth and pathogenicity [30]. The strong early immune response elicited by IHNV and other related RNA viruses has favored the development of several vaccines using a reverse genetics approach [32,33]; however, recently a DNA vaccine against IHN has been registered in Canada (Novartis Animal Health Canada, Inc).

LPS, the major constituent of the external layer of the outer layer of Gram-negative bacteria, is a widely used PAMP-preparation which induces potent immune responses in which the lipid A portion of the molecule is primarily responsible for the endotoxic properties observed in experimental animals [34,35]. Fish present a remarkable tolerance to LPS challenge in comparison to mammals which has been postulated to be due to differences in receptor-mediated recognition of LPS [36]. In vivo challenge to high concentrations of LPS in fish does not result in endotoxin-mediated mortality [23].

We have carried out *in vivo *challenges using either live or attenuated IHNV (infectious hematopoietic necrosis virus) or bacterial lipopolysaccharide (*E. coli *LPS) in trout (*Oncorhynchus mykiss*). Total RNAs from head kidney tissue were sampled, 1 and 3 days post intra-peritoneal injection (i.p.), and analysed by gene chip analysis. We have identified a generalised immune/stress/hematopoietic gene response to all treatments and a large set of viral-specific genes responding to IHNV infection. Gene ontology analysis presents two distinct physiological responses to either LPS or IHNV in which IHNV pathogenesis can be clearly identified. The response to LPS indicates a general inflammatory response followed by a significant hematopoietic response. Here we present a comparison of the differential gene expression patterns induced *in vivo *by a generic PAMP, *E. coli *LPS, and a viral pathogen, IHNV, and an attenuated form of the viral pathogen, attIHNV in the head kidney of the rainbow trout.

## Results and Discussion

### Fish survival and pathogenesis

The epizootiology of IHN in young fish has been thoroughly described [27,37-42] and includes widespread hemorrhages in kidney, liver and musculature leading to anemia, and extensive necrosis of major hematopioetic tissues (head kidney and spleen). No mortalities, external signs or histological lesions were observed in fish injected with IHNV or attIHNV at sampling time points. The remaining or non-sampled fish displayed the referred pathological features of IHN, arriving to a 100% mortality 7–9 days and 15–17 days after challenge with virulent or attenuated IHNV, respectively. In fish challenged with LPS no mortalities were recorded in experimental groups. This is a typical response in which fish do not show an appreciable 'septic shock' type response. A molecular mechanism has been proposed addressing LPS-tolerance in fish which may be due to differential signalling from Toll-like receptors in which the classical TLR4 paradigm differs from that observed in mammals. In fact, rainbow trout macrophages have been shown to be about 1000 times less sensitive to LPS than mammalian macrophages [9].

### Overview of viral and LPS-induced differential gene expression in the head kidney

In order to examine the transcriptional profile of head kidneys (HK) dissected from trout treated with either intra-peritoneal LPS or infection with IHNV or attIHNV we used a salmonid-specific cDNA microarray platform previously validated for studies involving stress, toxicity and immune response in trout [6-8,43].

**Figure 1:**
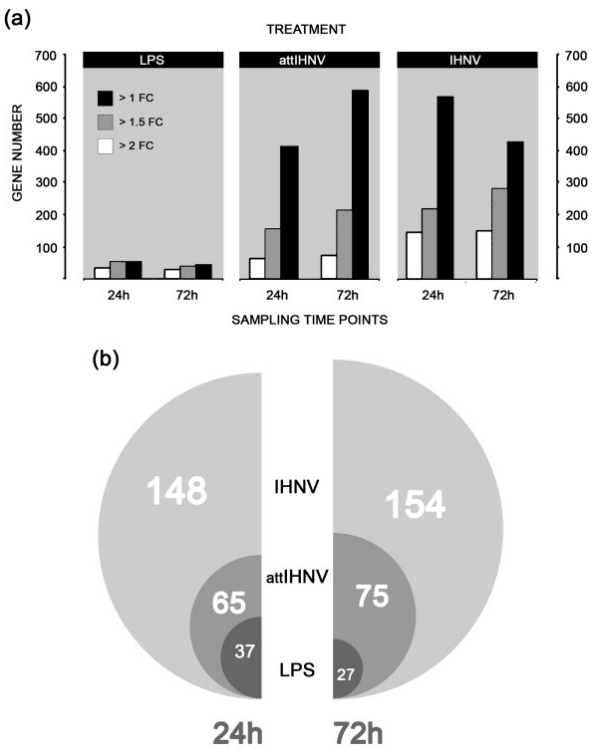
**Gene response to lipolysaccharide and viral (active and attenuated IHNV) in the head kidney of trout (*O. mykiss*)**. **(a) **Total gene number (>1 fold change; *p *< 0.01) expressed on the microarray in each treatment and in each time point **(b) **Differentially expressed genes (p < 0.01; FDR < 0.05) with a 2-fold increase in expression levels are shown for each treatment and time point.

Total numbers of genes significantly expressed in each of the treatments is shown in fig. 1 and a list of 20 selected genes, ranked by the expression levels was included in tables 1, 2, 3. Applying a selection criteria based in a classical cut-off value of >2 fold change over the differentially expressed genes (*p *< 0.01) emphasizes the stronger global induction of gene expression following the inoculation of both active and attenuated forms of IHNV (fig. 1b). Within differentially expressed gene groups (>1 fold change, *p *< 0.01; fig. 1a), bacterial LPS induces a higher response in comparison to viral groups. Although the number of ranked selected genes (*p*-value < 0.01) is considerably lower than in viral treatments, about 56.5% (n = 58) and 60% (n = 45) of regulated genes at 24 and 72 hours respectively achieve more than 2 fold change in its expression levels, in contrast with attenuated (15%; n = 413 at 24 h and 25%; n = 591 at 72 h) or active (26%, n = 570 at 24 h and 35.5%; n = 428 at 72 h) viral inoculation of IHNV.

**Table 1:** List of top 20 up and down regulated genes in the head kidney of trout, 24 and 72 hours after i.p. injection of LPS. Genes were selected for significant differential expression at *p *< 0.01 (FDR < 0.05). Values are expressed as FC, fold change.

**24 hours UP**				**72 hours UP**			
**Clone ID**	**Clone Name**	**FC**	***p***	**Clone ID**	**Clone Name**	**FC**	***p***
est03d11	Unknown-42	8.42	0.000	est03d11	Unknown-42	3.81	0.000
est04b01	Similar to rRNA (Vangl2)	4.54	0.004	est03e02	Hypothetical-fish 15	3.39	0.001
EXOB1_H05	Transposase-6	3.16	0.000	est04e04	MHC class 1b antigen	3.17	0.006
EXOB2_D06	NADH dehydrogenase subunit 5-1	2.76	0.000	EXOB2_H06	High affinity immunoglobulin epsilon receptor alpha	2.73	0.003
est02g07	Ig mu heavy chain disease protein	2.73	0.001	CA387837	Nucleolar protein NAP57	2.27	0.000
est02a11	Ig kappa chain V-IV region B17-2	2.71	0.002	EXOB4_C11	High affinity immunoglobulin gamma Fc receptor I precursor	2.15	0.008
EST1-3A_G09	Unknown-75	2.32	0.001	ENH2_F09	Beta-2-microglobulin-1	1.99	0.004
CA383564	Coatomer epsilon subunit 1	2.25	0.001	EXOB2_A09	60S ribosomal protein L5-2	1.80	0.005
est04e04	MHC class 1b antigen	2.24	0.007	HST0001_C05	Cell division control protein 42 homolog	1.72	0.009
EST1-3A_F12	Transposase-59	2.12	0.001	EXOB3_F09	MHC class I heavy chain-1	1.71	0.001
HK0002_D09	Prothymosin alpha	2.03	0.003	est01g04	5-aminolevulinate synthase	1.58	0.010
est02e09	Ig heavy chain V-III region HIL	2.00	0.005				
EXOB4_D12	Hpa repeat-1	1.99	0.001				
HK0003_E06	DnaJ homolog subfamily C member 9	1.96	0.005				
HK0003_E08	Unknown-194	1.90	0.007				
est04g12	Envelope protein	1.90	0.004				
HK0002_H08	TATA-binding protein associated factor 2N	1.79	0.003				
CA387665	Cytochrome P450 2J2	1.79	0.000				
est01h01	Unknown-19	1.76	0.009				
est03b11	Acidic leucine-rich nuclear phosphoprotein 32 A-1	1.68	0.008				

**24 hours DOWN**				**72 hours DOWN**			

EXOB2_D07	Ependymin related protein-1	-2.28	0.002	est04c10	Unknown-56	-2.04	0.000
HST0001_C04	Hemoglobin alpha chain	-2.42	0.004	CA367764	Calmodulin-1	-2.13	0.001
EST1-3A_D08	Glyceraldehyde-3-phosphate dehydrogenase-6	-2.49	0.009	est03c04	Matrix metalloproteinase-9	-2.21	0.000
EXOB1_H06	CC chemokine SCYA110-2	-2.55	0.005	HKT0001_E07	Actin, alpha skeletal 1	-2.28	0.002
EST1-3A_H06	Transcription factor jun-B-1	-2.89	0.003	est01f03	Deltex protein 1	-2.37	0.001
est03f08	Transaldolase	-2.91	0.002	est01e02	Thymosin beta-4-1	-2.73	0.002
EXOB2_H01	Unknown-100	-2.91	0.002	EXOB3_G05	Actin, cytoplasmic 2	-2.78	0.000
est02f08	Serine protease-like protein-1	-2.97	0.000	EST1-3A_D08	Glyceraldehyde-3-phosphate dehydrogenase-6	-3.14	0.000
EST1-3A_A09	Serine protease-like protein-2	-3.96	0.003	EST1-3A_B03	Hypothetical-fish 44	-3.16	0.007
CA371363	Glucose-6-phosphate isomerase-1	-4.35	0.000	CA348284	CCAAT/enhancer binding protein beta	-3.22	0.003
HST0001_D08	Beta-globin	-4.46	0.000	CA374193	Chemokine receptor CXCR4	-3.71	0.000
est04e05	Glutathione peroxidase-gastrointestinal	-4.54	0.000	utu04f08	Actin, alpha skeletal 5	-3.81	0.001
est01c04	Unknown-11	-6.17	0.001	KVkm2_F01	Unknown-224	-4.21	0.000
EXOB2_D05	Matrix metalloproteinase 9-2	-6.63	0.000	est01f01	DNA-binding protein inhibitor ID-1	-4.64	0.000
est01e10	Tolloid-like protein (nephrosin)-1	-6.69	0.001	est02f08	Serine protease-like protein-1	-4.64	0.001
CA348284	CCAAT/enhancer binding protein beta	-7.36	0.006	EST1-3A_A09	Serine protease-like protein-2	-5.52	0.000
est03c04	Matrix metalloproteinase-9	-7.44	0.000	EXOB3_H01	Matrix metalloproteinase-13	-5.88	0.007
EXOB2_G12	Tolloid-like protein (nephrosin)-2	-9.26	0.000	EXOB2_H01	Unknown-100	-12.08	0.000
EST1-3A_B03	Hypothetical-fish 44	-14.01	0.000	est01e10	Tolloid-like protein (nephrosin)-1	-12.97	0.000
EXOB3_H01	Matrix metalloproteinase-13	-17.18	0.000	EXOB2_G12	Tolloid-like protein (nephrosin)-2	-13.47	0.000

**Table 2:** List of top 20 up and down regulated genes in the head kidney of trout, 24 and 72 hours after i.p. injection of attIHNV. Genes were selected for significant differential expression at *p *< 0.01 (FDR < 0.05). Values are expressed as FC, fold change.

**24 hours UP**				**72 hours UP**			
**Clone ID**	**Clone Name**	**FC**	***p***	**Clone ID**	**Clone Name**	**FC**	***p***
EXOB2_B10	Hemoglobin beta chain	19.46	0.000	est03e08	Hypothetical-fish 36	3.06	0.000
HST0001_C04	Hemoglobin alpha chain	11.19	0.000	CA343473	C3a anaphylatoxin chemotactic receptor	2.98	0.000
KVkm2_A01	Unknown-219	10.93	0.000	EXOB2_G11	Stanniocalcin-1	2.93	0.000
HST0001_D04	Alpha-globin I-2	10.93	0.000	EXOB1_B02	Hypothetical-fish 34	2.84	0.000
EXOB4_A03	Carbonic anhydrase	10.57	0.000	KVkm2_H07	Unknown-226	2.77	0.000
CA366564	Huntingtin	8.52	0.000	EXOB3_A01	Unknown-103	2.73	0.000
EXOB2_B12	RING finger protein 103	8.34	0.000	CA363723	Cyclin C	2.64	0.000
HST0001_C07	Unknown-213	7.60	0.000	EXOB4_H01	Unknown-132	2.63	0.000
ENH2_E07	Unknown-3	7.04	0.000	est04c02	Unknown-53	2.61	0.000
EXOB4_G09	Histone H14	6.94	0.000	HK0002_G04	Fructose-bisphosphate aldolase A	2.45	0.000
EXOB4_H06	Alpha-globin 1-3	6.63	0.000	EST1-3A_H03	Unknown-76	2.42	0.000
utu01e09	Embryonic alpha-type globin2+collagen alpha 2(1)	4.94	0.000	CA358107	Ectonucleoside triphosphate diphosphohydrolase 1	2.42	0.000
HK0002_G02	Creatine kinase, sarcomeric mitochondrial precursor	4.49	0.000	CA378361	Ubiquitin ligase SIAH1	2.40	0.000
HK0001_C08	Galectin-9 (VHSV-induced protein)-3	4.14	0.000	HK0001_H12	Unknown-162	2.35	0.000
CA361101	Regulator of G-protein signaling 1-2	4.03	0.000	CA342204	TNF receptor associated factor 3	2.33	0.000
est02a11	Ig kappa chain V-IV region B17-2	3.19	0.000	EST1-3A_H05	Adenosine deaminase 3	2.31	0.000
CA343473	C3a anaphylatoxin chemotactic receptor	3.00	0.000	est04f10	Hypothetical-fish 1	2.30	0.000
est04b01	Similar to rRNA (Vangl2)	2.69	0.000	KVkm2_H11	Unknown-228	2.30	0.000
HST0001_D02	ATP-binding cassette, sub-family F, member 2	2.58	0.000	HKT0001_A11	Hypothetical-fish 11	2.28	0.000
CA384134	G1/S-specific cyclin D2	2.56	0.000	CA355893	DnaJ homolog subfami.B member 2	2.26	0.000

**24 hours DOWN**				**72 hours DOWN**			

est01e10	Tolloid-like protein (nephrosin)-1	-2.60	0.000	EXOB2_G02	Profilin-1	-2.13	0.000
est03c04	Matrix metalloproteinase-9	-2.70	0.000	EXOB2_F12	60S ribosomal protein L7a-1	-2.13	0.000
HST0001_C03	Plasminogen precursor-2	-2.72	0.000	est02g11	Cytochrome c oxidase subunit I-1	-2.14	0.000
KVkm2_H10	Unknown-227	-2.76	0.000	HK0001_H03	60S ribosomal protein L26	-2.14	0.000
CA34828	CCAAT/enhancer binding protein beta	-3.05	0.000	HK0003_A12	Heterogeneous nuclear ribonucleoprotein A1-1	-2.16	0.000
EXOB2_D05	Matrix metalloproteinase 9-2	-3.07	0.000	EXOB1_H12	NADH dehydrogenase subunit 4	-2.23	0.000
est02f08	Serine protease-like protein-1	-3.21	0.000	HK0002_D07	Unknown-172	-2.24	0.000
EST1-3A_A09	Serine protease-like protein-2	-3.25	0.000	EXOB3_G03	60S acidic ribosomal protein P2	-2.31	0.000
HK0003_C11	Tropomyosin alpha 3 chain-1	-3.34	0.000	EXOB2_G09	Cytochrome c oxidase subunit I-2	-2.34	0.000
utu03e06	Parvalbumin alpha-3	-3.50	0.000	HST0001_D04	Alpha-globin I-2	-2.37	0.000
CA370329	Lysozyme C precursor	-3.90	0.000	ENH2_H06	Unknown-5	-2.46	0.000
HK0003_C08	Parvalbumin alpha-2	-3.99	0.000	est04c05	Ferritin heavy chain-1	-2.54	0.000
utu04h1	Myosin light chain 2-2	-4.13	0.000	P_46	ATPase 6	-2.56	0.000
utu02c02	Myosin heavy chain, skeletal, adult 1-1	-4.30	0.000	utu02b07	Cytochrome c oxidase subunit II	-2.64	0.000
Hete0002_A07	Metallothionein-IL	-4.49	0.000	KVkm2_H10	Unknown-227	-2.67	0.000
HK0003_E07	Myosin light chain 2-1	-4.49	0.000	EST1-3A_H07	Cytochrome b-1	-2.86	0.000
HK0002_D07	Unknown-172	-5.04	0.000	est02h09	Nonhistone chromosomal protein HMG-17	-3.07	0.000
EXOB1_A03	Metallothionein A	-5.05	0.000	CA370329	Lysozyme C precursor	-3.08	0.000
est01c04	Unknown-11	-6.17	0.000	EXOB1_C02	Unknown-83	-3.44	0.000
HK0002_F05	Myosin heavy chain, skeletal, fetal	-7.31	0.000	EXOB2_G01	Leukocyte cell-derived chemotaxin 2	-3.56	0.000

**Table 3:** List of top 20 up and down regulated genes in the head kidney of trout, 24 and 72 hours after i.p. injection of active IHNV. Genes were selected for significant differential expression at *p *< 0.01 (FDR < 0.05). Values are expressed as FC, fold change.

**24 hours UP**				**72 hours UP**			
**Clone ID**	**Clone Name**	**FC**	***p***	**Clone ID**	**Clone Name**	**FC**	***p***
CA343473	C3a anaphylatoxin chemotactic receptor	5.35	0.000	HST0001_D04	Alpha-globin I-2	15.48	0.000
est04c02	Unknown-53	4.26	0.000	est02a11	Ig kappa chain V-IV region B17-2	10.06	0.000
est02b02	PEST-containing nuclear protein	4.00	0.000	EXOB4_D02	Beta actin-2	9.60	0.000
EXOB3_A01	Unknown-103	3.91	0.000	EXOB2_B10	Hemoglobin beta chain	9.58	0.000
CA358107	Ectonucleoside triphosphate diphosphohydrolase 1	3.69	0.000	HK0003_A03	Thymosin beta-4-2	7.56	0.000
EXOB4_H01	Unknown-132	3.63	0.000	est02f08	Serine protease-like protein-1	6.41	0.000
HK0002_G04	Fructose-bisphosphate aldolase A	3.59	0.000	est02h09	Nonhistone chromosomal protein HMG-17	6.00	0.000
EXOB1_B03	Unknown-81	3.59	0.000	est03c04	Matrix metalloproteinase-9	5.91	0.000
EXOB4_E09	Hypoxanthine-guanine phosphoribosyltransferase	3.55	0.000	est01e06	Coronin-1B	5.51	0.000
CA378361	Ubiquitin ligase SIAH1	3.43	0.000	est02g07	Ig mu heavy chain disease protein	5.44	0.000
HK0003_G05	Unknown-201	3.43	0.000	EXOB4_H05	Unknown-133	5.24	0.000
est04f10	Hypothetical-fish 1	3.41	0.000	est01e02	Thymosin beta-4-1	5.17	0.000
EST1-3A_G03	Unknown-74	3.40	0.000	EXOB3_D02	Eukaryotic translation elongation factor 1 alpha 1	4.60	0.000
CA378435	Protein phosphatase 2C delta isoform	3.35	0.000	EXOB2_A01	MHC class II invariant chain-like protein 1	4.44	0.000
HK0002_H10	Unknown-182	3.33	0.000	EXOB3_H01	Matrix metalloproteinase-13	4.34	0.000
EXOB1_F02	Transcription regulator protein BACH1	3.28	0.000	EXOB1_A05	Ribosomal protein L6-1	4.33	0.000
est04b01	Similar to rRNA (Vangl2)	3.26	0.000	EST1-3A_F05	Heat shock 70 kDa protein 1	4.27	0.000
EST1-3A_H05	Adenosine deaminase 3	3.21	0.000	EXOB2_G0	Profilin-1	4.00	0.000
HK0001_H12	Unknown-162	3.05	0.000	HKT0001_H03	Microtubule-associated protein RP/EB	3.95	0.000
CA368716	Membrane-bound transcription factor site 2 protease	3.00	0.000	CA370329	Lysozyme C precursor	3.91	0.000

**24 hours DOWN**				**72 hours DOWN**			

utu01a03	40S ribosomal protein S3-1	-3.22	0.000	EXOB1_B02	Hypothetical-fish 34	-4.48	0.000
HKT0001_H03	Microtubule-associated protein RP/EB	-3.24	0.000	HK0002_G04	Fructose-bisphosphate aldolase A	-4.80	0.000
utu04g05	40S ribosomal protein S9-3	-3.27	0.000	CA384134	G1/S-specific cyclin D2	-4.93	0.000
EST1-3A_D03	60S ribosomal protein L23	-3.32	0.000	EST1-3A_H03	Unknown-76	-5.18	0.000
EXOB1_H08	ADP, ATP carrier protein 3	-3.43	0.000	est03f04	F-box/WD-repeat protein 11	-5.28	0.000
EST1-3A_F05	Heat shock 70 kDa protein 1	-3.54	0.000	CA368716	Membrane-bound transcription factor site 2 protease	-5.31	0.000
EXOB1_A03	Metallothionein A	-3.55	0.000	CA384029	Chromobox protein homolog 4	-5.47	0.000
EXOB2_G09	Cytochrome c oxidase subunit I-2	-3.55	0.000	EXOB3_A01	Unknown-103	-5.54	0.000
HST0001_C03	Plasminogen precursor-2	-3.56	0.000	EXOB4_H01	Unknown-132	-5.99	0.000
EXOB3_E01	Na/K ATPase alpha subunit-2	-3.76	0.000	EST1-3A_H05	Adenosine deaminase 3	-6.23	0.000
HK0001_D01	Ubiquitin	-3.83	0.000	EXOB1_B03	Unknown-81	-6.29	0.000
HK0002_B07	Heat shock 70kDa protein 8	-4.24	0.000	HK0001_H12	Unknown-162	-6.44	0.000
Hete0002_A07	Metallothionein-IL	-4.50	0.000	HK0002_H10	Unknown-182	-6.59	0.000
utu02a08	Ubiquitin and ribosomal protein S27a-2	-4.43	0.000	est04c02	Unknown-53	-6.62	0.000
est02a11	Ig kappa chain V-IV region B17-2	-4.84	0.000	EXOB4_E09	Hypoxanthine-guanine phosphoribosyltransferase	-6.63	0.000
utu02c02	Myosin heavy chain, skeletal, adult 1-1	-4.97	0.000	est02b02	PEST-containing nuclear protein	-6.85	0.000
utu04f11	40S ribosomal protein S3-2	-6.00	0.000	est04f10	Hypothetical-fish 1	-7.01	0.000
est03c10	Alanine-glyoxylate aminotransferase 2	-7.41	0.000	EST1-3A_G03	Unknown-74	-7.55	0.000
est01c04	Unknown-11	-8.11	0.000	CA378361	Ubiquitin ligase SIAH1	-7.82	0.000
HK0002_D07	Unknown-172	-8.74	0.000	EXOB2_G11	Stanniocalcin-1	-9.69	0.000

**Figure 2:**
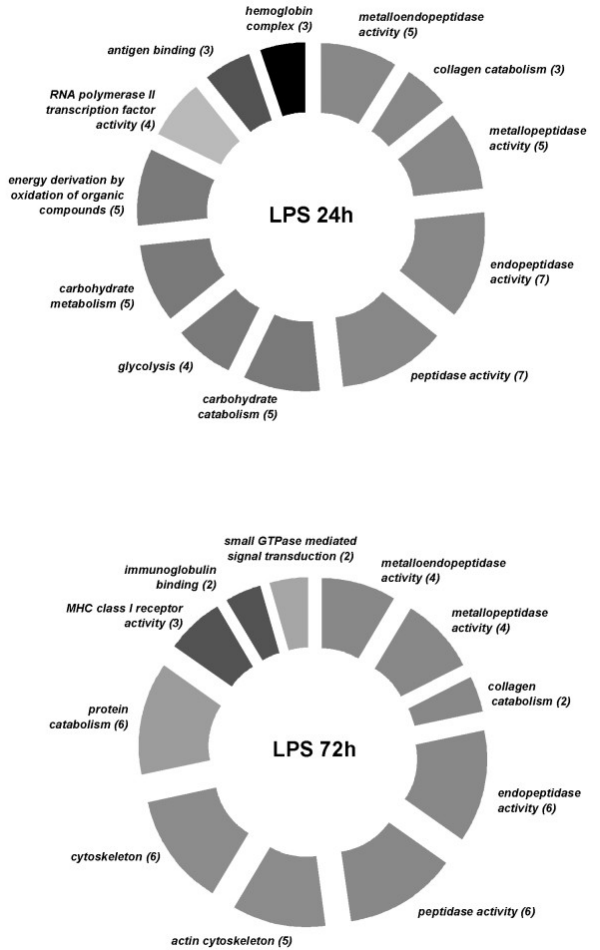
**Over represented GO functional classes in LPS array experiments**. Functional categories with Yates corrected Chi squared *p *< 0.05 were selected. Number of regulated genes for each category is shown in parenthesis.

**Figure 3:**
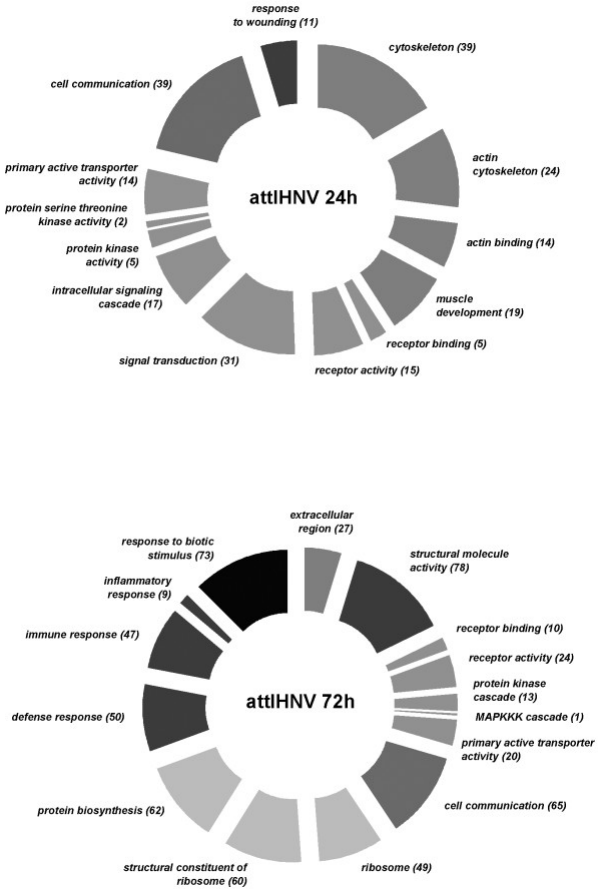
**Over represented GO functional classes in attIHNV array experiments**. Functional categories with Yates corrected Chi squared *p *< 0.05 were selected. Number of regulated genes for each category is shown in parenthesis

**Figure 4:**
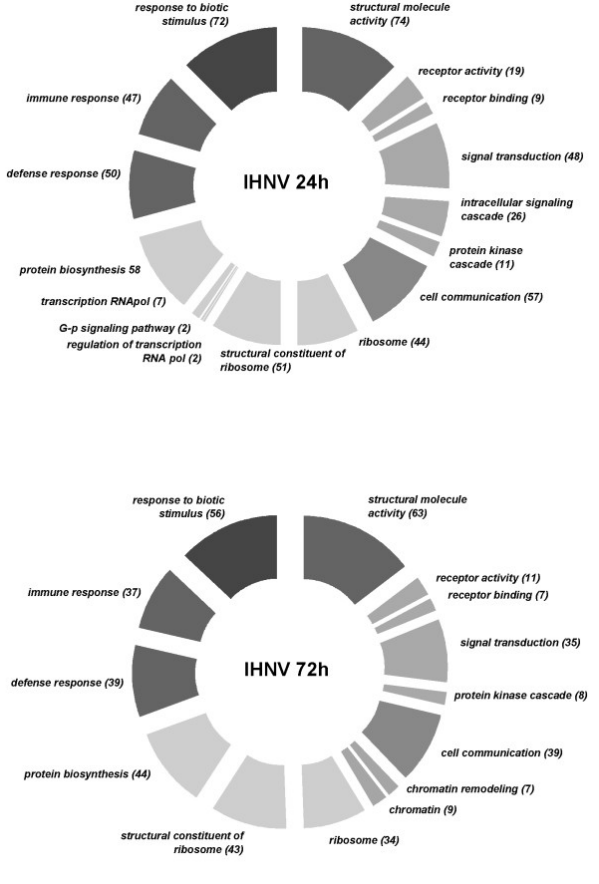
**Over represented GO functional classes in IHNV array experiments**. Functional categories with Yates corrected Chi squared *p *< 0.05 were selected. Number of regulated genes for each category is shown in parenthesis.

**Figure 5:**
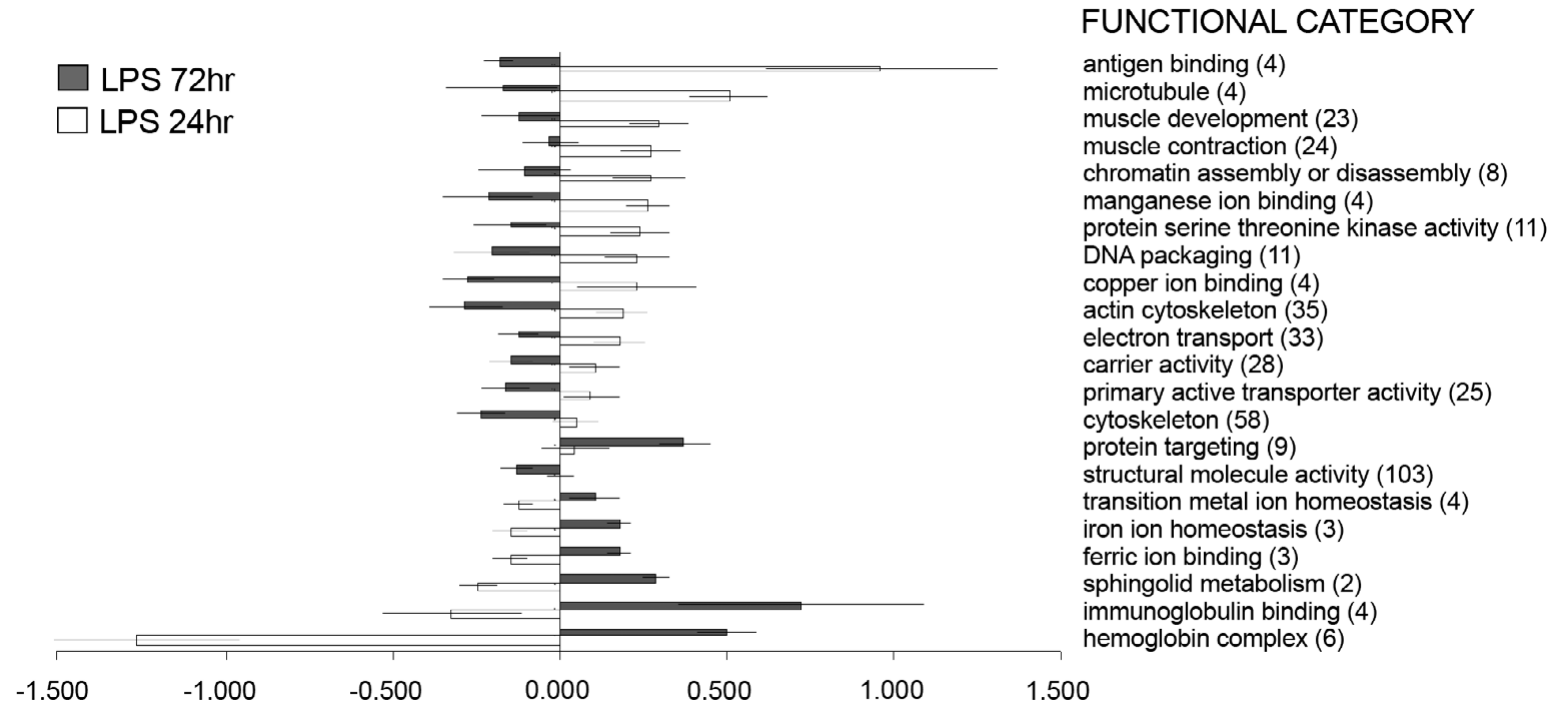
**Gene Ontology analysis for LPS treatment**. Results show categories with significant difference, (pair comparison independent test, *p *< 0.05), chosen from categories which had a p-value < 0.01 from individual treatment groups. Data is shown as mean fold change ± std.error.

**Figure 6:**
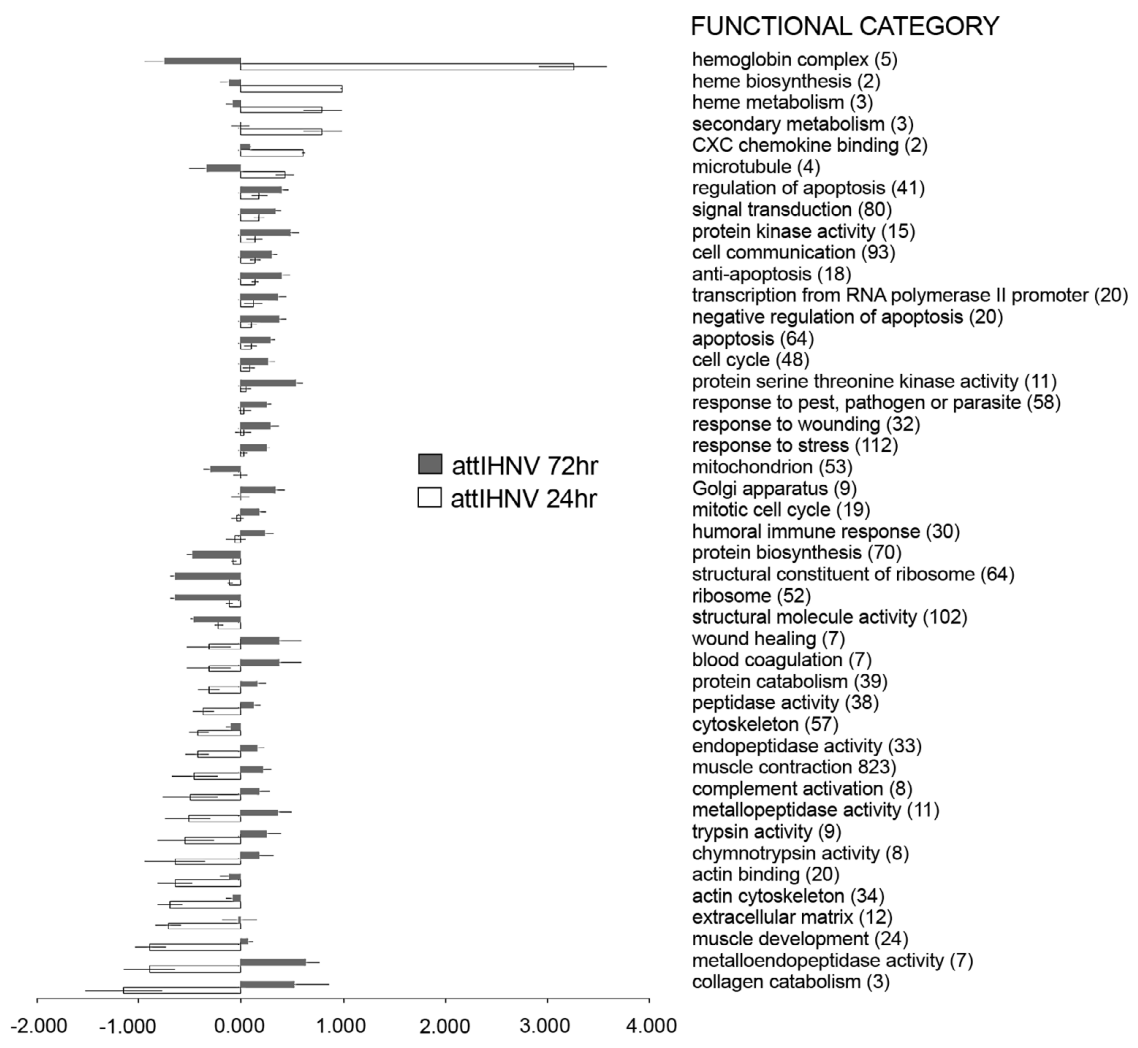
**Gene Ontology analysis for attIHNV treatment**. Results show categories with significant difference, (pair comparison independent test, *p *< 0.05), chosen from categories which had a p-value < 0.01 from individual treatment groups. Data is shown as mean fold change ± std.error.

**Figure 7:**
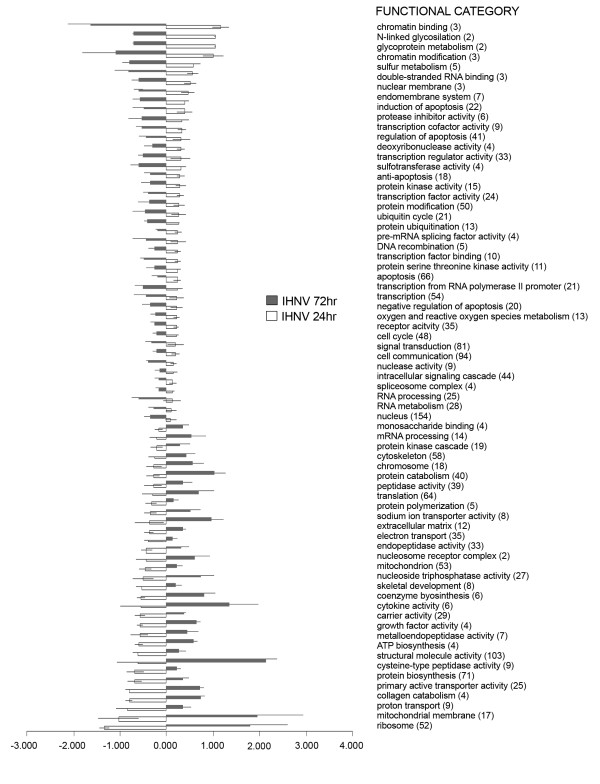
**Gene Ontology analysis for IHNV treatment**. Results show categories with significant difference, (pair comparison independent test, *p *< 0.05), chosen from categories which had a p-value < 0.01 from individual treatment groups. Data is shown as mean fold change ± std.error.

However, using the aforementioned selection criteria (>2 fold change; *p *< 0.01) the magnitude of the transcriptomic response, measured as the number of differentially expressed genes, shows a clear difference between active viral treatment and LPS, the former eliciting an extensive immune, apoptotic and transcriptional response (see the analysis of functional classes below; figs. 2, 3, 4, 5, 6, 7). As shown by the gene representation of the two viral treatments mirroring strength of induction at 1–1.5 fold change levels, transcriptomic responses also include an extensive repertoire of genes expressed below 1.5 fold with *p *< 0.01 that clearly outnumber the 2-fold expressed genes (Fig. 1a) and can be ascribed to low-level transcriptional, metabolic and homeostasis maintenance programs (see tables 4, 5 and figs. 2, 3, 4, 5, 6, 7). Whilst it may be expected that an attenuated form of the virus does not induce a similar magnitude of response, the species-specific onset of the immune response elicited by the highly antigenic viral glycoprotein (G) of attIHNV may undoubtedly contribute to the observed gene expression pattern, as described in several DNA vaccination assays for fish pathogenic rhabdoviruses [14,16,33].

**Table 4:** Genes common to all treatments. Genes selected were expressed in a minimum of 5 of the 6 experiments and compared against all experiments in the database (n = 155). Only genes specific to these experiments were chosen (Fischer's exact Test, *p *< 0.01). Values are expressed as FC, fold change.

**Clone ID**	**Clone Name**	**LPS (FC)**		**AttIHNV (FC)**		**IHNV (FC)**	
		**24 hr**	**72 hr**	**24 hr**	**72 hr**	**24 hr**	**72 hr**
CA384637	60 kDa heat shock protein-1	-	1.47	1.18	1.25	-1.25	1.29
est03b11	Acidic leucine-rich nuclear phosphoprotein 32 A-1	1.68	1.41	1.17	1.32	1.27	1.34
utu01f04	Actin, alpha skeletal 2	-	-1.31	-1.48	1.18	1.52	1.29
utu01g11	Actin, alpha skeletal 3	1.67	-1.64	-1.47	-1.25	1.34	1.42
utu04d04	Actin, alpha skeletal 4	2.00	-1.30	-1.18	-	1.61	1.37
EST1-3A_H05	Adenosine deaminase 3	1.57	-	1.14	2.31	3.21	-6.23
HST0001_D04	Alpha-globin I-2	-1.30	-	10.93	-2.37	-3.11	15.48
CA364941	Annexin A1-1	-2.08	-1.78	-1.36	-1.46	-1.26	1.68
F_122	ARP2/3 complex 21 kDa subunit	-	-.192	-1.41	-1.23	-1.57	1.33
P_10	Aryl hydrocarbon receptor	1.35	-	1.32	1.80	1.47	-2.32
CA367764	Calmodulin-1	-	-2.13	-1.26	-1.29	-1.73	1.49
CA348284	CCAAT/enhancer binding protein beta	-7.36	-3.22	-3.05	-	-2.16	1.76
CA363762	Cell death activator CIDE-B	1.32	-	1.35	1.74	2.08	-2.53
CA383564	Coatomer epsilon subunit 1	2.25	-	1.20	1.43	1.24	1.53
HK0003_G01	Creatine kinase, M-2	-	1.61	-2.02	1.32	-1.54	-1.40
HK0002_G02	Creatine kinase, sarcomeric mitochondrial precursor	-1.68	-	4.49	-1.54	-1.82	1.20
CA358998	Cysteine-rich protein 1	-1.75	-2.71	-1.37	-1.51	-1.62	2.75
utu01g04	Cytochrome oxidase subunit III-2	-	-1.36	-2.55	-2.04	-2.08	2.33
EXOB1_C10	Cytochrome P450 2K4-2	1.60	1.34	1.20	-1.44	-	-1.14
utu01d06	Deoxyribonuclease gamma precursor	1.50	-	-1.19	-1.26	1.46	-1.31
utu01e09	Embryonic alpha-type globin2+collagen alpha 2(1)	-1.62	1.42	4.94	-1.41	-1.96	-
HK0001_D12	Estrogen-responsive B box protein	1.29	-	-1.22	-1.14	2.11	-1.38
HK0002_G04	Fructose-bisphosphate aldolase A	1.65	-	1.25	2.45	3.59	-4.80
HK0001_A11	Fumarate hydratase, mitochondrial precursor	-	-1.58	-1.12	-1.21	1.43	-1.25
CA371363	Glucose-6-phosphate isomerase-1	-4.35	-1.68	-1.40	-1.15	-1.37	2.81
EXOB3_H05	Glucose-6-phosphate isomerase-2	-2.07	-	-1.51	-2.08	-1.77	1.81
CA366403	Heat shock 27 kDa protein-1	1.44	-	1.21	1.61	2.49	-2.43
HST0001_C04	Hemoglobin alpha chain	-2.42	-	11.19	-1.82	-2.93	2.87
EXOB2_B10	Hemoglobin beta chain	-1.86	-	19.46	-2.07	-2.45	9.58
EXOB4_C11	High affinity immunoglobulin gamma Fc receptor I precursor	-	2.15	-1.70	-1.73	-1.88	1.71
est02a11	Ig kappa chain V-IV region B17-2	2.71	-	3.19	-2.07	-4.84	10.06
est02g07	Ig mu heavy chain disease protein	2.73	-	2.38	1.56	-2.14	5.44
EXOB2_D05	Matrix metalloproteinase 9-2	-6.63	-2.62	-3.07	1.71	-1.93	3.20
EXOB3_H01	Matrix metalloproteinase-13	-17.18	-5.88	-1.32	2.06	1.44	4.34
est03c04	Matrix metalloproteinase-9	-7.44	-2.21	-2.70	1.68	-2.92	5.91
EXOB3_F09	MHC class I heavy chain-1	-	1.71	-1.43	-1.20	-2.87	-3.02
P_4	Myeloperoxidase	1.55	-	1.23	1.88	2.32	-3.19
EXOB3_E09	N-myc downstream regulated protein-2	-1.48	1.67	1.16	1.21	-	-1.32
EXOB2_G02	Profilin-1	-	-2.04	-1.79	-2.13	-2.12	4.00
est02f08	Serine protease-like protein-1	-2.97	-4.64	-3.21	1.32	-1.84	6.41
EST1-3A_A09	Serine protease-like protein-2	-3.96	-5.52	-3.25	1.32	-2.91	3.75
HK0002_H08	TATA-binding protein associated factor 2N	1.79	-	-1.32	-1.22	1.71	-1.32
est01e02	Thymosin beta-4-1	-	-2.73	-2.03	-1.32	-1.47	5.17
est01e10	Tolloid-like protein (nephrosin)-1	-6.69	-12.97	-2.60	-1.07	-3.11	2.98
EXOB2_G12	Tolloid-like protein (nephrosin)-2	-9.26	-13.47	-1.67	1.44	-1.75	3.70
Hete0002_E09	Transposase-56	2.06	1.96	1.42	1.30	2.04	1.67
EST1-3A_F12	Transposase-59	2.12	1.62	1.28	1.23	-	1.20
EXOB3_F11	Ubiquitin-conjugating enzyme E2-18 kDa	-	1.47	1.48	1.68	1.61	-1.84

**Table 5:** Genes specific to viral treatments. Genes selected were expressed in a minimum of 3 of the 4 experiments and compared against all experiments in the database (n = 155). Only genes specific to these experiments were chosen (Fischer's exact Test, *p *< 0.001). Values are expressed as FC, fold change. *13 Unknowns were removed*.

**Clone ID**	**Clone Name**	**AttIHNV (FC)**		**IHNV (FC)**	
		**24 hr**	**72 hr**	**24 hr**	**72 hr**
CA387866	Arachidonate 5-lipoxygenase-2	1.16	1.34	1.27	1.03
CA384555	B-cell-specific coactivator OBF-1	1.48	1.76	1.37	-1.09
CA343473	C3a anaphylatoxin chemotactic receptor	3.00	2.98	5.35	-1.50
ENH2_F03	Cathepsin K-1	-2.30	1.22	-3.01	1.42
EXOB4_F03	Cathepsin S	-1.46	-1.28	-1.65	2.80
CA376117	CD231	1.31	1.55	2.04	-1.66
CA363762	Cell death activator CIDE-B	1.35	1.74	2.08	-2.53
EXOB4_E08	Cytochrome c oxidase subunit VIIa-related	1.08	-1.14	1.18	-2.47
CA381440	Double-stranded RNA-specific adenosine deaminase	1.45	1.53	1.69	-1.67
CA351392	Guanine nucleotide exchange factor DBS	1.19	1.93	2.97	-3.13
EXOB4_C11	High affinity immunoglobulin gamma Fc receptor I precursor	-1.70	-1.73	-1.88	1.71
HKT0001_A11	Hypothetical-fish 11	1.20	2.28	1.49	-1.85
est03c07	Ig kappa chain V-I region WEA	1.60	2.07	1.41	1.21
est02g07	Ig mu heavy chain disease protein	2.38	1.56	-2.14	5.44
CA363978	Inhibitor of kappaB kinase gamma	1.16	2.13	2.21	-2.36
EXOB3_F09	MHC class I heavy chain-1	-1.43	-1.20	-2.87	-3.02
CA341859	Nuclear factor NF-kappa-B	1.40	1.12	1.63	1.28
CA378435	Protein phosphatase 2C delta isofor	1.44	2.05	3.35	-3.87
EXOB1_B07	PRPF39 protein	1.19	1.22	1.39	
est03e10	Putative inorganic polyphosphate/ATP-NAD kinase	-1.11	-1.05	1.33	1.29
est03b12	Secretory granule proteoglycan core protein	-1.83	-1.83	-2.97	2.77
ENH2_B08	Splicing factor, arginine/serine-rich 8	1.10	1.24	1.25	-1.49
CA380121	Telomerase reverse transcriptase	1.33	1.36	1.34	-1.33
EXOB1_G12	Thioredoxin	-1.24	-1.61	-1.56	1.46
CA342204	TNF receptor associated factor	1.23	2.33	2.64	-2.89
CA378736	Tyrosine-protein kinase BTK	1.28	1.61	1.81	-1.80
EXOB3_G04	Tyrosine-protein kinase HCK	-1.07	-1.38	-1.39	1.40
P_54	VEGF4	1.23	1.34	1.37	

Not surprisingly, both viral treatments display a 10% increase in the number of genes regulated >2 fold at 72 hours post-treatment. Previous studies on the pathogenicity of IHNV in salmonids typically describe a 24 h delay in detectable viral titres and internal measures of immunological disturbance, followed by a rapid increase in the differential expression of viral-related and acute phase response genes in major haematopoietic organs and liver, respectively [42,44]. In addition, a tissue-specific effect determines the dynamics of the transient cellular populations during inflammation and, subsequently, the tissue-dependent gene expression profile. It has been suggested [45] that the transient double stranded RNA intermediates produced by the accelerated replication of IHNV 24 hours post infection seem to regulate the expression of trout Toll-like Receptor 3 (rtTLR3) in a unpredictable manner, strongly dependent on viral growth and host lymphocyte recirculation cycles. Interestingly, the pattern of rtTLR3 expression in response to *Yersinia ruckeri*, a gram-negative bacterial trout pathogen shows a remarkably lower magnitude in terms of fold change compared with the viral challenge [45], mimicking the magnitude of the response between LPS and IHNV/attIHNV treatments described above (fig 1b). Although both treatments display increases in the number of regulated genes the patterns of gene expression are also significantly different. The number of genes regulated show that LPS induces in the majority a down-regulation of gene expression at both time points. Similar results were observed in LPS-stimulated macrophages derived from trout, *O. mykiss*, analysed with the same platform [8]. Highest fold changes in individual genes were observed in down-regulated genes in the LPS groups (table 1). On the other hand, viral treatments induce a higher induction of transcriptomic and cell cycle/apoptotic activity where induction and suppression processes display a similar weighting (tables 2 and 3).

The viral treatments show the highest differential gene expression counts obtained when compared across all available experiments in our gene (KuopioChip) database. This observation, together with the extensive transcriptional onset observed in the analysis of functional ontology categories in viral treatments (see below), follows that of Abbas *et al*. (2006) suggesting that a specific immune response mobilizes more transcriptional remodelling than the majority of physiological responses.

### Specific vs. common gene responses

From the selected differentially expressed genes (*p *< 0.01) in LPS and viral treatments, the differential response in LPS and viral treatments (significant differential expression in at least 5 of the 6 experiments) was evaluated across 155 experiments in our gene database (KuopioChip) in order to ascertain those genes co-expressed (Fischer's exact P < 0.01) in each or both experimental conditions (minimum requirement was significant differential expression in at least 5 of the 6 experiments). A total of 49 genes were identified from which many genes showed opposite responses representative of directional responses to viral and LPS-induced stimuli (table 4 and 5).

Interestingly genes regulated by the attIHNV treatment show a mixture of effects both similar and different to LPS and IHNV suggesting that different mechanisms, non-specific and specific response, of activation are induced by the attenuated virus in the head kidney. Alpha globin for example shows down regulation by LPS whereas attIHNV initially induces expression, 11 fold, followed by suppression, -2.37 fold. On the other hand IHNV causes suppression at 24 hours followed by strong induction, 15 fold. LPS and attIHNV show similar profiles for: Glucose-6-phosphate isomerase, annexin 1, the nephrosin proteins, calmodulin-1, CEBP-beta, cysteine-rich protein-1, cytochrome oxidase and cytochrome p-450. The majority of the genes are related to immunity and the inflammatory response. Suppression of HK proteases, nephrosin and MMPs, seems to be one of the most characteristic effects of LPS (Nephrosins FC; 24 hours; -6.69 and -9.26 and 72 hours -12.97 and -13.47) whereas viral treatment causes a similar initial effect followed by induction of expression (FC at 72 hours; attIHNV 1.44 and IHNV 2.98 and 3.7). Serine protease-like proteins 1 and 2(spl1 and spl2) are also highly suppressed by LPS, attIHNV and IHNV at 24 hours. This suppression continues in the LPS treatment, however in viral samples we observe a significant increase in expression in the 72 hour samples (attIHNV; 1.32 both spl1 and 2, IHNV; 6.4 and 3.75). Several cytoskeleton related genes including ARP2/3, the actin and profilin-1 are down-regulated by both LPS and attIHNV whereas IHNV induces up-regulation of all genes in this group at 72 hours post-infection.

Viral treatments induce a typical adaptive immune response (table 5). The antigen processing/presenting loading pathways include the differential expression of MHC Class I and lymphoid and myeloid cell lineages, as shown by the regulation of B-cell specific coactivator OBF-1, essential for the response of B-cells to antigens and required for the formation of germinal centers, the conservation of BTK B-cell, HCK neutrophil and TNFalpha receptor signaling pathways, the cathepsin mediated antigen processing and the interferon-inducible RNA-specific adenosine deaminase ADAR1 (table 5).

Not surprisingly, NF-kappa-B (NFkB) signal transduction suffered from moderate to severe regulation in the head kidney of infected trout (tables 3 and 4). NFkB is considered a pleiotropic transcription factor expressed in several cell types undergoing amongst others inflammatory assaults. Both active IHNV and attIHNV treated fish showed differential regulation of Inhibitor of kappaB kinase gamma, member of a family of proteins which inactivate NFkB by trapping it in the cell cytoplasm [46] and are actively expressed in virus infected cells [47].

The anaphylactic arm of the complement activation acts in a double fashion through the component C3a/C3aReceptor: as a chemotactic mediator involved in endotoxic responses and, simultaneously, as a regulator of homing and mobilizations of hematopoietic stem cells (see below). Both complement related serine proteases (spl) and C3 proteolytic components were differentially expressed in LPS-induced or viral treatments. However, the highest levels of complement expression seem to be strongly dependent of viral invasions in fish head kidney (tables 3 and 5)

### Stromal protease activity and extracellular matrix remodelling in trout head kidney

Although the precise function of nephrosin, an astacin metalloproteinase [48] remains unresolved, it has been shown to be involved in the late stages of granulocyte differentiation and cell migration and/or tissue infiltration processes in challenged carp and zebrafish [49,50]. In trout treated with LPS, the two nephrosin proteins genes are severely down regulated (between -6 and -10 fold) at both time points (fig. 1). The rest of metalloproteinases (MMP 9-2, 13) also showed a marked decrease of expression in LPS treated trout (less than -6 fold change) at both time points and at 24 h in IHNV or attIHNV infected fish (-1.3 to -3 fold; see table 2 and additional file 1), therefore suggesting a decrease in extracellular matrix remodeling and leukocyte movement.

In mammals, MMP 9 has been implicated in the signal processing and maturation of dendritic cells, IL-8 mediated activation of neutrophils and undifferentiated hematopoietic stem cells [51,52]. Whilst the existence of functionally differentiated dendritic cells in fish is still controversial [53,54], the mammalian leukocyte response outlined for IL-8 and MMP 9 seems to be conserved in teleosts, although the exact pattern of organ distribution, the intensity of gene regulation and the voluble species-specific expression of IL-8 in fish HK remains obscure [55,56]. The chemotatic properties of IL-8 on leukocytes are amplified by the recruitment of neutrophils mediated by CXC chemokines. Our data show a moderate (-3.71 fold change) down-regulation of trout CXCR4. Mammalian CXCR4 forms with TNFalpha, several HSP proteins and GDF5 an activation cluster involved in monocyte LPS signal transduction [57] and also acts synergistically with the colony-stimulating factor mediated mobilization of hematopoietic stem cells [57,58].

The MMP gene family appears to be induced by LPS in trout macrophages [8], and a widescreen transcriptomic analysis of carp metalloproteinases detected a large amount of MMP9 mRNA mainly in hematopoietic organs, HK and spleen [59,60]. Fish metalloproteinases thus probably act in a mammalian fashion, showing and up-regulation by the synergistic stimulation of Tumor Necrosis Factor alpha (TNFalpha) and LPS. In agreement with the low level expression of MMPs observed 24 h post injection, no significant amounts of TNFalpha mRNA were detected in LPS treated trout, in contrast with IHNV infected fish. Both myeloperoxidase, a lysosomal hemoprotein characteristic of mononuclear phagocytes, and TNF were up regulated 24 h post injection of attenuated IHNV in trout HK (2.3 and 2.6 fold, respectively). In the active viral group, however, TNFalpha was found to be inactive (see below). The moderate up-regulation of TNFalpha 3 days post attIHNV stimulation (table 2) may suggest, according to the well described model of macrophage stimulation by LPS [8], the onset of a proliferative myeloid response in the principal hematopoietic organ.

Intracellular thymosin beta-4 (TBX4) is considered the main G-actin sequestering peptide in mammals [61]. The putative functions of extracellular TB4X include induction of hemostasis, wound and tissue healing, chemotaxis, induction of metalloproteinases, granulocyte-mediated inhibition of inflammatory processes and regulation of hematopoietic stem cell proliferation [62]. Not surprisingly, the TBX4 gene is >2 fold down regulated in trout HK 72 h post injection of LPS (table 1), but strongly upregulated (>5–7 fold) in IHNV infected trout undergoing an incipient hemorrhagic symptomology. This suggests that, in fish as in mammals, the pleiotropic effects of TBX4 are mediated by the remodeling of intracellular actin cytoskeleton and/or components of the extracellular matrix [63].

The moderate/severe down-regulation of CXCR4 1 day post LPS challenge (-3.7 fold change), metalloproteinases and TB4X observed at 24 h and 72 h (less than -6 and -2 fold change levels, respectively; table 1) thus favors a scenario of delayed reactive state to LPS stimulation in the recirculation and traffic of trout head kidney hematopoietic cells following i.p. administration of bacterial LPS. Moreover, the head kidney itself shows a low level stromal extracellular matrix remodeling in trout treated with LPS, as suggested by the down regulated expression of actin, MMP, nephrosins and Thymosin beta 4 (table 1). However, at 3 days post IHNV infection the transcriptomic footprints in the head kidney reveal an immunological shift orientated toward a somewhat impaired adaptive arm activation coupled with a strong hemostatic and extracellular matrix sculpting response: the systemic spreading of IHNV clearly inhibits TNFalpha, MHC class I and several macrophage and cell cycle/differentiation markers (tables 3, 4; see below) favoring a MHC class II, immunoglobulin and MMP/TBX4 enhanced immune response.

### Complement response to LPS-induced and viral challenges in trout head kidney

Contrary to the liver, the head kidney cannot be considered an acute phase reactive organ. Following a xenobiotic assault, complement related serine proteases, modulators and C3 proteolytic peptides are synthesized primarily in the liver. However, minor but biologically significant extra-hepatic (mainly in active immune cells, gills, skin, heart, gonad and renal tissues) synthesis of complement components has recently been demonstrated in fish as in other vertebrates [64,65]. Therefore, complement inflammatory, chemotactic, opsonic and lytic activities extend the effect of the innate arm of immune responses to the core of major hematopoietic organs.

In trout injected with LPS, complement related serine proteases (spl1 and spl2) homologous to the MASP proteins involved in the activation of the classical complement pathway [66,67] remained down regulated throughout the treatment (-3 and -4 fold change at 24 h and 72 h respectively; table 1) and no expression of C3 genes were detectable in head kidney (table 1), in concordance with the biased adaptive response observed in this group (see below). A recent microarray analysis of acute phase reactivity in the liver of catfish (*Ictalurus punctatus*) showed an enhanced (greater than >2 fold) complement response in fish infected with *Edwardsiella ictaluri*, a gram negative pathogen responsible for enteric septicemia in catfish [13] In trout, Lovoll *et al*. (2007b) found a similar expression pattern in hepatocytes treated with LPS, but, in sharp contrast with the highest expression levels of complement genes in liver, a minor up regulation of C3 genes in head kidney and spleen was observed. Additionally, gene expression showed a strong tissue and isoform dependence: C3-4 was found to be down regulated in HK following stimulation with LPS, and more interestingly, not all trout isoforms maintained similar levels of gene expression [68].

The tolerance of fish to the standard LPS doses used in rodent immune challenges is well known, and has been linked to the peculiarities of PAMP receptors in fish [36] that may preclude a strong endotoxic shock response. Thus, the strongest cellular and tissue responses to i.p. injection LPS in fish are thought to be restricted to activated monocyte/macrophages and lymphocytes, portals of entry (gills, intestine, skin) and acute phase organs (liver). However, the *dynamics *of the teleostean immune response is of primary interest in these organs, as in the head kidney, that functions either as a cradle for immunological priming of leukocyte populations or as a major node in the complex and the still poorly understood network of neuro-immune-endocrine interactions in fish. In that respect, our results uncover a striking difference between lipopolysaccharide and viral treatments concerning head kidney transcriptomic dynamics: in attenuated and active IHNV groups, the complement related serine proteases and anaphylatoxin receptors (C3aR) maintain up regulated expression levels at both time points, with the maximal expression (>5–6 fold change) in active viral groups.

The C3aR shows an unequivocal upregulated expression at 24 h and 72 h in animals infected with attIHNV, and at 24 h in animals infected with active IHNV (our results also show a tenuous down regulation of C3aR (-1.5 fold change) in IHNV treated fish at 72 h; see additional file 1). At 72 h, the response to IHNV consist of a mixture of adaptive (MHC, IG) and innate (C3aR, complement related serine proteases, lysozyme C) immune and stress (HSP70, hemostasis) responses against a background of metalloproteinase-mediated matrix remodeling (table 3). Therefore, as suggested in this and previous studies with mammals [69-71], the expression of C3a/C3aR may probably contribute to the homing/mobilizations and differentiation of hematopoietic stem cells in response to the generalized immune and stress response elicited by an aggressive and extremely pathogenic virus in fish. Moreover, in mammals several C3 cleavage fragments, including C3a has been demonstrated to be linked to the CXCR4-mediated responsiveness of hematopoietic stem cells [71]. The moderate down regulation of CXCR4 in LPS treated fish (table 1) and upregulation (>1.5 fold) in attIHNV infected fish (see additional file 1), together with the strong upregulation of C3aR in IHNV treated fish suggests a conservation of complement mediated functional responses in the hematopoietic head kidney. The dynamics and trafficking of hematopoietic and differentiated cells in fish are, nonetheless, far from being fully understood.

### Specific immune responses to LPS-induced and viral challenges in trout head kidney

Genes involved in the immunoglobulin system increased in all treatments displaying different kinetics. Ig gamma Fc receptor (CD64) gene expression increased late in the LPS treatment whereas IgM heavy chain (B-cells) and Ig kappa chain V-IV region B17-2 (involved in antigen presentation) increased acutely. Under attIHNV conditions a similar regulation is observed for the latter genes in which IgM expression is sustained at 72 hours and Ig Kappa suppressed. CD64 expression is suppressed by attenuated virus, whereas IHNV suppresses all three genes at the early stage and induces expression (1.7, 10 and 5.4 fold respectively) at the later stage of infection. This may reflect migration of leukocytes to the primary sites of infection/inflammation and/or differential recruitment of leukocyte sub-populations to the infected head kidney in both IHNV/attIHNV treatments.

The coatomer protein (COP) epsilon known to play a role in the formation and maturation of phagosomes [72] is induced in all treatments, and the Class I major histocompatibility complex (MHC) antigen, well known to be involved in antigen presentation in dendritic cells is activated late by LPS and suppressed by viral treatments (-3 fold; tables 2, 3), whereas Class II MHC showed a moderate up regulation (4.4 fold) in fish infected with IHNV at 72 h.

In fish, as in mammals, the interaction of MHC molecules with T cell receptors (TCR) seems to activate subsets of cytotoxic T lymphocytes (CTL) and T helper cells (Th) in a similar fashion [73]. Homologues of mammalian MHC, several proteins associated to antigen presentation, beta2 microglobulin and CD8+ and CD4+ (markers for CTL and Th, respectively) have been recently characterized in trout and other fish species [74,75], thus reinforcing the conservation of antigen processing pathways in immune cells. However, the trafficking, recirculation and cell-to-cell communication against a quiescent/activated immune background have not yet been properly described in fish. Moreover, the translation of classical bacterial or viral inflammatory murine models encompasses several technical (full characterization of immune processes, absence/presence of mediators, conservation of activation/inhibition pathways) and species-specific difficulties, the latter related to the high variability of the interspecific thresholds of immune activation in fish and, last but not least, the relative virulence and co-evolutionary trade-offs of pathogens.

Our and several recent studies have attempted to resolve these issues weighting the organ related immune response to an established model of LPS-induced inflammation or viral infection. In this regard, the expression of CD8 and CD4 (coreceptors of MHC Class I and II binding, respectively) in trout seems to be restricted mainly to thymus and to a certain extent, to spleen, even though non-infected fish maintain a widespread low level expression in several hematopoietic or lymphocyte infiltrated organs [74,75]. Overturf and LaPatra (2006) were unable to find elevated levels of CD8 expression in the HK of trout infected with bacteria or IHNV at 24 h or 5 days post infection, although in liver and spleen a positive dose-response correlation followed the infection (24 h) expression of CD8 and C3 [76]. In a similar experiment, 72 hours after IHNV challenge Hansen and LaPatra (2002) observed a surprising tissue-specific shutdown of MHC Class IIB mRNA in head kidney and spleen of infected trout, thus suggesting an enhanced CD8 response coupled with activation of MHC Class I antigen presentation following IHNV infection [77], as observed in a cohabitant model of fish viral infection described recently [78]. Similarly, an infection by *Vibrio anguilarum *seemed to depress the short term (up to 4 days) expression of MHC class II genes in head kidney, liver and spleen of turbot, *Scophthalmus maximus *[79]. However, an elevated expression of MHC Class II genes has been described in trout following i.m. DNA vaccination with recombinant IHNV [80] and xenobiotic inflammation [81]. In trout challenged with VSHV, a member of Rhabdoviridae, the dynamics of T cell expansion, and thus the onset of MHC mediated adaptive immune response, were found to be correlated with the expected waves of viral replication, with peak a week after viral challenge [82], mimicking the delayed (up to 10 days) expression of MHC mRNAs that were also observed in japanese flounder (*Paralichthys olivaceus*) leukocytes infected with *Neoheterobothrium hirame*, a monogenean parasite [83].

As described in other species [84,85], our results suggest a predilection for CD4/Th lymphocyte response in the head kidney of trout challenged with IHNV at 72 post infection, coupled with a strong spl-induced complement cleavage activation and MMP/TBX4 extracellular matrix sculpting, together with a decreased TNFalpha mediated activation of monocyte/macrophage populations, a shutdown of MHC Class I and also a low level regulation of apoptosis, as shown by the inhibition of Ubiquitin ligase S1AH1 and Galectin-9 (see table 3 and additional file 1).

The i.p. administration of LPS activates the MHC Class I pathway of antigen processing in concordance with previous studies with LPS-activated trout macrophages [8]. However, the transcription factor CCAAT/enhancer binding protein-alpha (C/EBP-alpha), best known for its role in driving myeloid cells towards the granulocytic line [86] but also known to be induced during macrophage differentiation, and the MMP/TBX4 response was inhibited in head kidney 72 h post injection. Deltex protein 1 (DTX) also appears to be down regulated 72 h post infection with LPS (table 1). Although still poorly understood, the cross-talk between Deltex protein 1 (DTX1) and the evolutionary conserved Notch and NFkappaB signaling pathways [87,88] allows the normal development and maturation of differentiated lymphocyte populations in hematopoietic and lymphopoietic organs in mammals. This suggests an impaired trafficking of lymphoid/myeloid HK cells in the early response to LPS in trout.

Myeloperoxidase (MPO) was up-regulated by all treatments at 24 hours returning to baseline in LPS samples, increasing in attIHNV and actively inhibited in IHNV samples (tables 1, 2, 3, 4). Myeloperoxidase is expressed in neutrophils and monocytes and plays a role in the oxygen dependent mechanism of phagocytosis. The macrophage scavenger receptor MARCO [89] was also inhibited in IHNV samples. Therefore, the microbiocidal function was diminished in the head kidney of IHNV infected trout.

Taken together, these results may suggest a minor function of the head kidney in the *short term *(24–72 hrs) activation of the immune response to virulent IHNV, or, alternatively, an inducible and maybe antagonistic early differential expression of MHC Class I or II mediated antigen processing in the head kidney, heavily influenced either by the type and infective dynamics of pathogen (LPS vs. viral) or the portals of entry (organ/tissue). From the experimental infections described above, the liver and spleen also seem to act as a major acute phase reactive organs at the initial stages of viral invasion, and the head kidney can be more properly defined as an inductor of a delayed adaptive response as much as a major regulator of erythropoietic and myeloid differentiation. Nevertheless the dual expression of the MHC antigen processing/presenting machinery appears to be strongly influenced by the species-specific immunological sensibility [90,91], the virulence of pathogens and the timing of cellular differentiation in immune organs. This pathogen/host species-specific branching of MHC mediated immune response in fish requires, however, further analysis.

### Haemoglobin metabolism in LPS-induced and viral infected trout head kidney

Hemoglobin genes (α, β) are suppressed by both LPS and IHNV at 24 hours, 2–3 fold suppression, however attIHNV strongly induces expression of both proteins(11 and 19 fold respectively) followed by suppression at 72 hours. IHNV induces expression (3 and 10 fold α and β respectively) at 72 hours whereas LPS samples return to baseline levels.

Interestingly, 5-aminolevulinate synthase, the key enzyme involved in heme synthesis was up-regulated in both attIHNV samples and in a LPS-specific manner at 72 hours. A similar response has been observed in head kidney tissue from Atlantic salmon, *S. salar*, infected with Piscirickettsia [15].

The coordinated expression of both hemoglobin genes and 5-aminolevulinate synthase suggest erythropoietic activity in the head kidney. Furthermore, a significant increase in cellular proliferation in PU-1-ve cells, 72 hours post-LPS administration, in the head kidney of LPS-treated rainbow trout has been reported (Ribas et al, 2007 *in press*) thus activation of hematopoietic, potentially erythropoietic, mechanisms during the early stages of infectious processes may be a standard for the non-specific immune response in fish.

### HSP induced stress response to LPS-induced and viral challenges in trout head kidney

Active IHNV inhibits the expression of heat shock protein (HSP) 27 (-2.4 fold) and induces the expression of 70 and 90 (2.8 and 2.3 fold respectively) 3 days post infection from a previous inactive state at 24 h (-4.24 fold). The attIHNV showed a down regulation of HSP70 (-2 fold) at the same time point.

HSPs have been implicated in the generalized stress response associated to xenobiotics and/or inflammatory reactions in fish [44,85,92]. However, the reliability of HSP as an indicator of stress or pathologic/immune disturbances has recently thoroughly criticized [93] because of the great variability of measured HSP expression: despite the correlated expression of HSP with altered states in stressed or injured fish, the sensitivity and intensity of HSP response can vary in a species-specific manner, and among tissues, HSP families, season, developmental stages and stressor. Not being an acute phase response organ, it is, thus, difficult to speculate about the fate of HSP repaired enzymatic and/or cytoskeleton proteins in infected head kidney.

## Conclusion

For the functional analysis of biological roles of regulated genes, our two-step approach in the first instance establishes a list of differentially expressed genes whose ascribed biological roles are evaluated and secondly by identifying overrepresented GO functional categories using the KuopioChip analysis software (see materials and methods). This methodological approach is not exempt of limitations. The selected cut-offs for minimal gene expression, the co-expressed patterns of gene expression, the non lineal genome-proteome crosstalk and the limited transcript enrichment of the array can either limit the amount and quality of transcriptomic responses assessed or exclude transient but biologically relevant genetic responses correlated with the abruptness and organ-dependent systemic damage in infected fish.

As a guideline for elucidating the biological response of viral/LPS-induced challenge, the comparison of differentially expressed genes by the GO categories showed a marked induction of metalloproteinases and other collagen and extracellular matrix sculptors in LPS treated trout, coupled with a decrease in genes controlling the basal metabolism and an increase in the activity of immune related mediators of MHC antigen presenting and immunoglublin-mediated opsonisation (figures 2 and 5) 72 h post infection.

Several genes involved in signal transduction and protein biosynthesis were active in attIHNV groups 72 h post infection, following a decrease in cytoskeleton remodelling. As in active IHNV infected trout, the functional GO categories are strongly enriched in genes active during inflammatory, immune and defence responses. In both attenuated and active IHNV treated fish, the immune response at 72 h clearly outweighs the metalloproteinase orientated LPS response, but, as described above, the response of the trout head kidney transcriptome to IHNV infection was more robust and diversified in number and immune related activation pathways.

## Methods

### Animal protocol an experimental infections

Rainbow trout, *Oncorhynchus mykiss*, were obtained from two commercial fish farms. Fish were maintained in flow through tanks under ambient conditions of light (photoperiod 10L/14D) and temperature (15 ± 2°C). Fish were fed with commercial trout pellets *ad libitum *and acclimated for at least two weeks prior to use in experiments. The health status of the animals and water quality were checked on a daily basis. Challenge experiments were carried out separately for each treatment and were performed in recirculating tanks with aeration at 14°C. Trout (n = 10/tank) weighing between 70–100 g were separated into 6 different tanks, 3 control and 3 experimental tanks. For experimental stimulations the fish were mildly anaesthetized (MS-222, 40 ppm, stage II of anaesthesia according to Iwama et al. 1989) and intraperitoneal (i.p.) injections carried out. Fish were intraperitoneally injected with saline (control) and LPS (6 mg/Kg; serotype 026:B6, Sigma, #L-8274), or with 100 μl of a 10^6 ^pfu/ml dilution of IHNV or attIHNV or culture medium (negative control). At defined time points, 24 and 72 hours post i.p. injection, animals (total n = 6), control and experimental, were selected from each tank (n = 2) and sacrificed by over anesthetization (MS-222, 100 ppm, stage III of anaesthesia [94]). Head kidneys (HK) were immediately dissected out, pooled and processed for total RNA purification using Tri Reagent (Molecular Research Center, Cincinnati, OH, USA) according to the manufacturer's protocol [95,96].

### Viruses and cell line

IHNV (French isolate 32/87) was used and propagated in the fish epithelial cell-line EPC derived from common carp (*Cyprinus carpio*) [97]. EPC cells were cultured in Eagle's minimum essential medium (MEM, Gibco) supplemented with foetal bovine serum (FBS), penicillin (100 IU ml^-1^), streptomycin (100 μg ml^-1^), buffered with 7,5% sodium bicarbonate and incubated at 20°C. The virus was inoculated on EPC grown in MEM with antibiotics and 2% FCS at 14°C. When the cytopathic effect was complete, the supernatant was harvested and centrifuged to eliminate cell debris. The virus stock was titrated according to Reed & Muench [98] in EPC 96 well plates. Attenuated IHNV was generated by reverse genetic engineering of virulent IHNV by M. Bremont as described elsewhere [31].

### RNA isolation

RNA from head kidneys of experimental and control/sham-injected fish were extracted with the Trizol reagent (Life Technologies) or Tri Reagent (Molecular Research Center, Cincinnati, OH, USA) according to the manufacturer's instructions. DNase treatment was performed to remove contaminating DNA from preparations. RNA was precipitated using ethanol 100% and ammonium acetate (pH 5.2). The RNA was stored at -80°C in ethanol 70% until use.

### Microarray design and analyses

The design of the microarray is described in detail elsewhere [6,7] and a full description of the platform and data presented in this manuscript are accessible through the public GEO depositories (accession number GPL6155 and GSE10272). In brief, the platform included 1380 genes printed in six replicates each. Random clones from common and subtracted cDNA libraries (976) were compared with the known vertebrate proteins using blastx and 686 genes were identified; the functional annotations were transferred from the putative homologs. These clones were supplemented with 297 genes selected by the categories of Gene Ontology. Overall, each microarray was enriched in a number of functional classes, such as stress and defense response (145 and 105 genes, respectively), cell cycle (62 genes), signal transduction (114 genes), chaperone activity (41 genes), and apoptosis (79 genes).

Total RNA obtained from head kidney tissue was verified for quantity and integrity by denaturing electrophoresis and labeling with Cy3- and Cy5-dCTP (Amersham Pharmacia) was completed using SuperScript III reverse transcriptase (Invitrogen) and oligo(dT) primer, and cDNA was purified with Microcon YM30 (Millipore). We used a dye swap experimental design [99] and each sample was hybridized to two microarrays. For the first slide, test and control cDNA were labeled with Cy5 and Cy3 respectively, and for the second array dye assignment was reversed. All head kidney samples were analyses using the dye swap protocol. The slides were pre-treated with 1% BSA, fraction V, 5 × SSC, 0.1% SDS (30 min at 50°C) and washed with 2 × SSC (3 min) and 0.2 × SSC (3 min) and hybridized overnight in cocktail containing 1.3 × Denhardt's, 3 × SSC 0.3% SDS, 2.1 μg/μl polyadenylate and 1 μg/μl yeast tRNA. All chemicals were from Sigma-Aldrich. Scanning was performed with ScanArray 5000 and images were processed with QuantArray (GSI Luminomics). The measurements in spots were filtered by criteria *I*/*B *≥ 3 and (*I*-*B*)/(*S*_*I *_+ *S*_*B*_) ≥ 0.6, where *I *and *B *are the mean signal and background intensities and *S*_*I*_, *S*_*B *_are the standard deviations. After subtraction of mean background, locally weighted non-linear regression (Lowess) normalization [100] was performed separately for each slide. To assess differential expression of genes, the normalized log intensity ratios were analyzed with Student's t-test (*p *< 0.01). The Bayesian modification to the false discovery rate (FDR) was used to correct for multiple comparison tests, estimating the *q*-value for the set of differentially expressed genes [101]. For the analysis of the functional profiling of samples, all genes that showed significant differential expression (*p *< 0.01) in at least one sample were used. Due to the large number of genes, the statistical significance of over represented functional categories in each experiment (figs. 2, 3, 4) was assessed using the Yates correction to Chi square test (corrected *p *< 0.05). The log (*p*-level) ranked up or down-regulated genes were analyzed interrogating the functional classes of Gene Ontology (GO) [102] and compared by the sums of ranked genes (Student's t-test, p < 0.05). A list of LPS and virus specific responsive genes (figs 5, 6, 7) was obtained by GO data mining of a database (KuopioChip) of 155 previous immune and stress related experiments [7,43].

### Quantitative RT PCR

Primers were designed to amplify 194–305 bp fragments. RNA was processed with Rnase-free Dnase (Promega). Synthesis of cDNA with Superscript III reverse transcriptase (Invitrogen) was primed with oligo(dT). Analyses were carried out using Dynamo SYBR Green kit (Finnzymes) and ABI Prism 7700 (Amersham-Pharmacia)

In order to quantify mRNA expression, real time PCR (Q-PCR) was carried out. cDNA was diluted 1:50 for target mRNA and 1:100 for 18S and used as a template with the primers Q-PCR Fw and Q-PCR Rv (see additional file 1, table 7). Wells (20 μl final volume) contained 10 μl of iQ™ SYBR Green Supermix (Bio-Rad), 500 nM concentration of forward and reverse primers and 5 μl of cDNA. Controls lacking cDNA and controls containing RNA were included. Reactions were run in a MyiQ thermocycler (BioRad) under the following protocol: 5 min initial denaturation at 95°C, followed by 40 cycles of 10 sec denaturation at 95°C and 30 sec at 60°C, and a final melting curve of 81 cycles (from 55°C to 95°C). All samples were run in triplicate and fluorescence was measured at the end of every extension step. C_T _(threshold cycle) values for each sample were expressed as "fold differences", calculated relative to untreated controls and normalized for each gene against those obtained for 18S (see additional file 1, Table 8).

## Authors' contributions

AF, BN, SM, JCB, and LR performed the experimental viral and LPS stimulations. AK carried on the microarray hybridizations and quality control. NR performed the qRT-PCR. SM and JCB performed the statistical analysis of all microarray data sets and drafted the manuscript. All authors read and approved the manuscript.

## Supplementary Material

Additional file 1 **IHNV-LPS trout microarray design and genes**. Design of microarray platform. List of genes selected for significant differences at 24 and 72 hours in LPS, attIHNV and IHNV treatments. QPCR validation of specific clones Click here for file

Additional file 2 **Microarray sequence clones**. List of annotated sequence clones. Click here for file
